# Protect Me, Lord, from Oil, from Water, from Fire, and from Ants and Save Me from Falling into the Hands of Fools

**DOI:** 10.3201/eid1305.000000

**Published:** 2007-05

**Authors:** Polyxeni Potter

**Affiliations:** * Centers for Disease Control and Prevention, Atlanta, Georgia, USA

**Keywords:** Indian painting, Indian miniatures, illustrated books, antimicrobial drug resistance, the Citadel of Naraka, Krishna storms the Citadel of Naraka, Krishna, art and science, art commentary, about the cover

**Figure:**
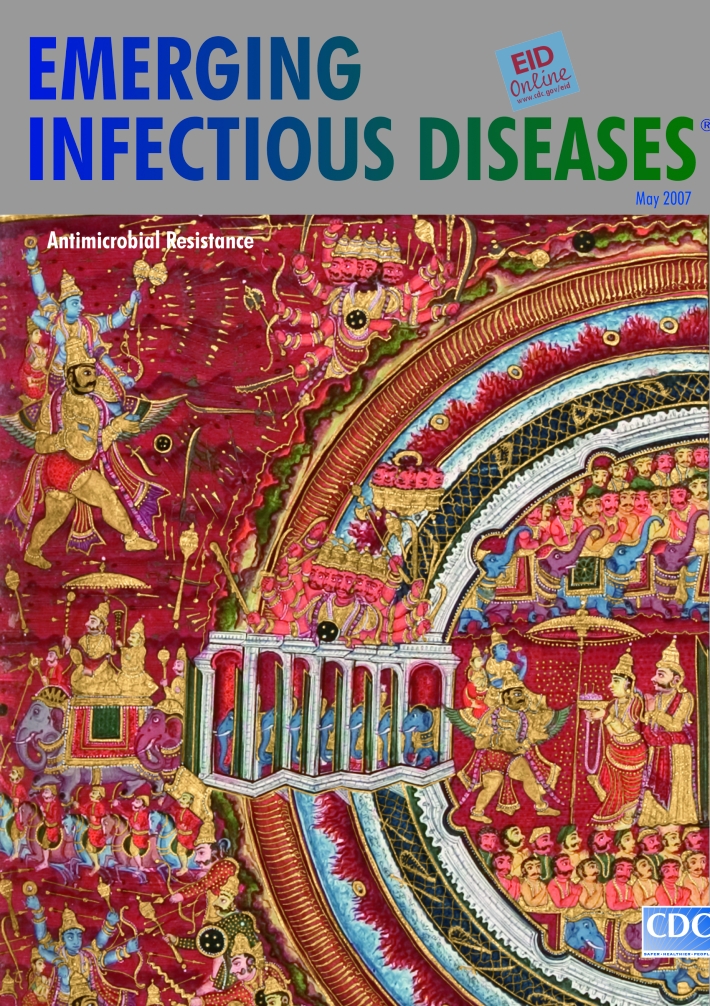
**Krishna Storms the Citadel of Naraka (from a Bhagavata Purana). India, Karnataka, Mysore (ca. 1840).** Opaque watercolor and gold on paper (25.1 cm × 36.8 cm). San Diego Museum of Art (Edwin Binney 3rd Collection)

– Prayer “uttered by a manuscript.” Found at the end of medieval Indian texts

Indian paintings on paper, known as “miniatures,” can be found in books from as far back as the 11th century, most from the 14th through the 19th century. They vary from postage stamp size to more than a yard in height and are called miniatures partly to distinguish them from murals, which they followed as a genre (*1*). Like the good books they inhabited, they were portable and intimate, meant to be appreciated from close up and, duly treasured, they were tucked away to be handled only from time to time, with care.

Miniature paintings were collaborative, created by groups of artists specialized in drawing, portraiture, background, or border illustration and were exclusively commissioned by patrons—princes, merchants, religious leaders. The importance attached to patronage can be traced in the colophons of surviving books. We know virtually nothing about the anonymous artist who created the painting but can often trace at whose “lotus feet” it was placed when completed.

Though “All the blessings of heaven” were bestowed on the patron of a manuscript or series, great patrons did not emerge until the late 15th century. Soon after, during the Mughal Empire, interest in art peaked, along with patronage, and schools of painting developed and flourished.

Miniatures were often painted on a wash: sheets of paper glued together and laminated. Ground white chalk or lead formed the foundation for layers of transparent watercolor in vivid, exotic pigments, from gum arabic or crushed seeds of the tamarind. Indian yellow was made of dried urine from cows fed on mango leaves. Gold, in leaf or liquid, embellished clothing and jewelry. Detail was created laboriously with fine brushes of hair from live squirrels, luster achieved from burnishing the surface, which also bonded pigment layers to the support (*2*). Book pages were intricately illustrated, some double-sided, with calligraphic elements on the verso.

The folio on this month’s cover comes from the Bhagavata Purana, a celebrated text in Hindu sacred literature recited daily by millions. Though favored and revered by painters and patrons, the Purana, with its collection of “ancient and wondrous tales of the Lord” Krishna, has rarely been illustrated with such exuberance (*1*). The embroidered cover of this manuscript, which contains 217 paintings, identifies it as volume 6 in a series. It was written on European paper. A seal on the flyleaf reads, “His Highness, Rajah of Mysore.”

Eyes are naturally drawn to Krishna. His name literally means “black” or “dark” or “all-attractive,” and he has a very distinct iconography. In his countless avatars, from Vishnu to simple human, his beauty is irresistible, his complexion “tinged with the hue of blue clouds” (*3*). Clad in golden silk, he rides the sun-bird Garuda. The philosophy of this God/cowherd is captured in the epic of the Hindu faith, the Bhagavad-Gita (*4*).

Krishna Storms the Citadel of Naraka recounts the God’s exploits against a demon king, a menace who commits atrocities, even against his own mother, the Earth Goddess. Aboard Garuda with his consort Satyabhama, Krishna wings his way to the demon’s citadel, “Which heart would not quail at the loud blast …from the Lord’s conch?” (*1*). The enemy is barricaded in his impregnable island city, inaccessible by “hilly fortifications and mounted missiles and weaponry” and unapproachable with “moats of water and fire and belts of stormy winds” (*1*).

Krishna, in true form, is Vishnu, four-armed and impervious to “thousands of fearful and strong snares” (*1*). He faces Mura, the five-headed demon (upper right), who soon falls, “like a mountain summit struck by a thunderbolt.” Mura’s seven sons move in, advancing, “discharging volleys of shafts, swords, maces, darts, double-edged swords and javelins” to perish too, along with their armies (*1*). Naraka joins in and succumbs to Krishna, who appears everywhere, “like a cloud emblazoned in a streak of lightning” (*1*). The citadel is penetrated. Inside, the Earth Goddess, bowing, offers Krishna “a pair of earrings resplendent with jewels and chased in the purest gold…a garland of forest flowers, the umbrella of Varuna…” (*1*).

The unfolding spectacle encompasses the heavens, engaging with ease gods, humans, animals, and mythologic beasts. Tiny figures move about purposefully, elephants carry on with dignity, seas are alive with fish. The monumental story is painted with assurance, as if it could have happened only in this orderly and brilliant way. And flying arrows and severed heads notwithstanding, the event seems a pageant, the celebration of a shift in the balance of power, an interaction whose outcome was never in doubt.

The citadel of Naraka with its formidable fortifications and hordes of defenders begs an equivalent in the microbial world. And not only because vermin threaten everything, even books. In the eternal, complicated interactions between microbes and hosts, supremacy and survival are closely knit. Host defenses are inevitably overcome by adaptation and change, until more sophisticated, specialized defenses can be built. Microbes develop resistance. Hosts mount additional defense. Microbes regroup and reappear in manifestations and avatars rivaling those of Krishna himself.
